# From Root Cause Analysis to Systems Thinking: A Comparative Content Analysis of Patient Safety Incident Investigation Reports in Mental Healthcare

**DOI:** 10.1111/jep.70495

**Published:** 2026-06-07

**Authors:** Alexander Challinor, Sahil Bhandari, Oladayo Bifarin, Esmaeil Khedmati Morasae, Panchu Xavier, Pooja Saini, Kathryn Berzins, Rajan Nathan

**Affiliations:** ^1^ Research and Development, Mersey Care NHS Foundation Trust Liverpool England UK; ^2^ Primary Care and Mental Health, Institute of Population Health Sciences University of Liverpool Liverpool England UK; ^3^ Person Centred Complex Care National Institute for Health Research Applied Research Collaboration North West Coast Liverpool England UK; ^4^ School of Psychology Liverpool John Moores University Liverpool England UK; ^5^ Senior Research Leader Programme National Institute for Health and Care Research (NIHR) Hertfordshire England UK; ^6^ Business School University of Strathclyde Glasgow Scotland UK; ^7^ Applied Health Research Hub University of Central Lancashire Preston England UK; ^8^ Chester Medical School University of Chester Chester England UK; ^9^ Research and Development, Cheshire and Wirral Partnership NHS Foundation Trust Chester England UK

**Keywords:** complexity, incident, psychiatry, root‐cause analysis, safety, system

## Abstract

**Rationale:**

The National Health Service, UK, has recently implemented a new patient safety strategy, replacing root cause analysis (RCA) incident investigation with systems‐based approaches. It is unknown if this change will optimise learning and improve care outcomes.

**Aims Objectives:**

We aimed to analyse safety recommendations/actions/improvements/solutions from comprehensive incident investigations by comparing those that adopted root cause analysis with systems‐based approaches.

**Method:**

The evaluation adopted a sequential multi methods design. Reports were extracted between January 2022 and January 2023. The quality of the incident investigation was graded using a validated tool (Learning Response Review and Improvement Tool). Investigation identified solution types were organised using qualitative content analysis, adopting inductive and deductive orientations. These were then classified into system factors and the effectiveness of the solution scored. Descriptive statistics were computed to investigate differences between incident investigation type.

**Results:**

Grading the quality of reports demonstrated that the expectations set out within the change in safety strategy were mostly being realised in practice. A total of 135 solutions were extracted from systems‐based and 57 from RCA reports, where the type of solutions identified were similar between each investigation approach. Organisational system factors were the most frequent for systems‐based whilst task system‐work factors were most frequent for RCA reports. For both investigation types, most of these solutions were deemed to fall in the least effective category: administrative controls.

**Conclusion:**

The evaluation provides important insights into how the shift to systems‐based investigations are shaping the quality of investigations and the recommendations that aim to prevent a recurrence of harm. Changing from RCA to systems‐based investigations led to more patient/carer/family involvement and systems‐focussed solutions, however weaker administrative recommendations remained prominent. Policy, practice and research need to ensure that the change in conceptual thinking and investigative orientation also contributes to improvements in learning and the development of stronger controls or barriers that prevent harm.

## 1 Introduction

The goal of the patient safety system in healthcare is to reduce the impact and incidence of avoidable harm [1]. When potential and/or actual harm does occur, the safety system in healthcare classifies this as a patient safety incident. Healthcare services have invested heavily in building a resilient incident response system that enhances efficiency, prevents harm recurrence, improves patient outcomes, and accounts for the complexity of safety systems [2, 3]. Incident investigations aim to identify factors causing harm, providing insights to improve services and prevent future incidents [4]. A critical strategic shift has occurred in patient safety in the National Health Service (NHS), England, United Kingdom (UK), where a systems‐based approach to investigation is now advocated, encouraging a more constructive view of patient safety that addresses systemic factors rather than isolating individual fault [5, 6]. Despite this shift, a recent review of system‐based approaches to safety in mental healthcare showed that there has been limited empirical study of a link between an investigation and the process of implementing evidence‐based improvements to reduce harm [7]. Thus, research is needed to evaluate the outcomes of systems‐based approaches to incident investigation and the processes that link investigation, learning and sustainable change.

Systems‐based approaches explore how parts of a system interact to achieve collective goals [8, 9]. Healthcare is a complex adaptive system with multiple levels that continually adapt to cope with any variability in the system. Safety within the healthcare system is an emergent property of this adaptive system, where risk can not only arise from individual parts but also from their relationships and interconnected components of the system in ways that are difficult to predict even when in possession of knowledge about the components [10]. Traditionally, safety was viewed through linear cause‐and‐effect relationships, with incident investigation determining safety by its absence (i.e., when things go wrong), rather than its presence [10, 11]. This is known as the Safety‐I approach. In contrast, Safety‐II emphasises learning from everyday *work‐as‐done* rather than imagined scenarios of learning/improvement from adverse incident investigation (*work‐as‐imagined)*. The NHS safety strategy now integrates both Safety‐I and Safety‐II, reflecting a more comprehensive approach to managing risk and fostering resilience [5, 12].

In March 2020, NHS England launched the Patient Safety Incident Response Framework (PSIRF), replacing the Serious Incident Framework (SIF) [5]. Previously, under the SIF, an initial incident review was requested where there was concern about a level of harm identified. This review would determine whether it met the threshold of a serious incident, an event where learning achieved from an investigation would be significant, or the consequences to patients, families, carers, staff or organisations are substantial enough to require further investigation. Under the SIF, all serious incidents required further investigations following the initial review. The serious incidents were escalated through a tiered investigation process: initial 72 h review, a level one internal investigation for serious and complex incidents, and level two with external review for more complex cases. For serious, complex cases, a comprehensive internal investigation report was required to be completed within sixty working days.

PSIRF shifted focus towards system‐based investigations, moving away from root‐cause analysis. Patient Safety Incident investigations (termed Patient Safety Learning Response Review (PSLR) Report in the studied organisation) are now guided by national mandates and local priorities [5]. As such, not all serious incidents above a certain threshold require a comprehensive investigation. This framework allows for more selective incident investigations, which facilitates a more proactive approach to learning with greater resources to focus on improvement than investigations [5]. PSIRF endorses the use of the Systems Engineering Initiative for Patient Safety (SEIPS) framework for systems analysis of incidents and safety issues [5]. The SEIPS framework, inclusive of the work system model, consists of six broad work factors: external environment, organisation, internal environment, tools and technology, tasks, and person(s) [5]. Patient safety incidents may result from multiple interactions between these work system factors.

Research into incident management systems shows there are shortcomings with traditional Root Cause Analysis (RCA) approaches. Participants in one study expressed dissatisfaction, criticising the RCA methodology as inadequate for driving meaningful learning and improvement [13]. Studies evaluating RCA outcomes show that problems are far more frequently identified than actionable solutions, and when solutions are proposed, they often lack evidence of effectiveness [14, 15]. One major factor appears to be the inherent complexity of systems, which limits RCA's ability to generate actionable insights. A study evaluating how incident data is used to improve safety found that incident review meetings primarily focussed on sharing practical, immediate information, while offering little reflection on systemic issues, causality of harm, or the implementation of corrective measures [16]. Another study showed that although incident reporting was a powerful tool to develop awareness of risks, converting those reports into tangible care improvements is highly challenging due to the intricate structure and processes of healthcare organisations [17].

In mental healthcare, the challenge is even greater. Research has argued that RCA approaches are misaligned with the nature of mental health incidents. Traditional RCA incident investigations attempt to impose linear cause‐effect thinking onto complex, context‐dependent events [18]. The causation behind mental health events is often uncertain, rendering the root‐case conjectural [19]. The complexity of services is compounded by the unpredictability of patient risks, making the effective application of incident investigation to drive safer care particularly difficult [17]. This has been demonstrated by RCA investigations producing weak, generic recommendations, which have attributed issues to human error and focussed on what is possible rather than what is needed [19, 20].

The shift from RCA to systems‐based approaches presents a promising opportunity to address persistent challenges in mental healthcare safety. Qualitative interviews with professionals embedded within safety systems indicate that this shift is widely perceived to enhance learning from investigations, foster improvement and strengthen safety culture [15]. Nevertheless, reservations persist regarding the actual efficacy of these safety investigations in delivering tangible improvement to patient care and in mitigating the risk of future harm [15]. At present, there remains a dearth of robust evidence demonstrating that systems‐based approaches yield more rigorous evidence‐informed and systems‐oriented safety solutions in comparison to the conventional RCA approach. A method of capturing the real‐world impact of the shift from RCA to systems‐based lies in the systemic evaluation of investigation reports generated following serious incidents.

To the authors' knowledge, there have been limited studies evaluating solutions generated from systems‐based incident investigations in healthcare. By comparing incident investigations that adopt differing methodological approaches (RCA or systems‐based), we can assess if this change generates more actionable insights capable of driving meaningful change across clinical practice, organisational policy and system‐level safety. Additionally, evaluating one healthcare organisation's responses to the changes in safety strategy may identify areas of good practice and help develop sustainable mechanisms for embedding learning within complex adaptive safety systems.

### 1.1 Aims

The aim of this evaluation was to investigate the recommendations arising from patient safety incidents investigations in one organisation. To achieve this, our objectives were to: (i) comparatively analyse the solutions obtained from safety incident investigation reports for both systems‐based patient safety incident investigations (under PSIRF) and Root Cause Analysis‐based internal investigation reports (under SIF); (ii) grade the quality of the investigation reports; (iii) rank the effectiveness of recommendations to prevent future harm.

## 2 Methods

### 2.1 Study Design

The study was a sequential multi‐methods design. Initially, we graded the quality of each patient safety report using a validated tool, the Learning Response Review and Improvement Tool, a tool designed to provide constructive feedback on the quality of safety investigation reports, developed by research and validated with users of the tool [5]. The tool consists of eight questions (see Table 1) that cover key areas to review providing structure to review an incident investigation report. The key domains are the purpose and focus of the review, the methodology to gather and analyse data (systems‐based using appropriate tools), the involvement of patient and family, the identification of system factors (rather than human error), the development of recommendations that are system focussed, and the clarity and structure of the report [5].

**Table 1:** Average score of investigation reports using the Learning Response Review and Improvement tool rating scale.

Area of review		Score (mean*/s.d)*		
No.	Descriptor	SIF/RCA	PLSR	*p*‐value
1	People affected by incidents are compassionately engaged and meaningfully involved	0.86 (0.69)	2.0 (0)	< 0.001*
2	A systems approach is used to investigate	1.14 (0.69)	2.0 (0)	< 0.001*
3	'Human Error' is considered as a symptom of a system problem	0.86 (0.9)	1.43 (0.53)	0.174
4	Blame language is avoided	0.86 (0.89)	1.57 (0.53)	0.096
5	Local rationality is considered	1.42 (0.53)	1.86 (0.37)	0.108
6	Counterfactual reasoning is avoided	1.0 (2.0)	1.57 (0.53)	0.207
7	Safety recommendations are systems‐focussed, evidence based and were developed collaboratively	1.0 (0.82)	1.86 (0.38)	0.027*
8	The written report is clear and easy to read	2.0 (0)	2.0 (0)	—
Total	—	1.09 (0.41)	1.73 (0.52)	—

*Note:* Rated as 0 = little evidence, 1 = some evidence, 2 = good evidence.

*Significance level set at < 0.05.

We then conducted a qualitative content analysis of the recommendations extracted from incident investigation reports, which involved three steps. First, the range and nature of investigator‐identified solutions were examined and categorised into solution types. Second, the solutions were categorised into key systems‐based work factors as defined within the SEIPS framework [5, 21]. Finally, we rated the effectiveness of the recommendations using the hierarchy of risk control measure, described further below [22]. Descriptive statistics were used to calculate the frequencies of factors within and between investigation reports.

The study followed the reporting guideline for the Reporting of Studies Conducted using Observational Routinely‐collected Health Data (RECORD) Statement [23]. The research reporting guideline checklist is found in material SA.

### 2.2 Study Setting

The study took place within one mental health NHS trust in England, UK. A mental health NHS trust is an organisation that provides health and social care services for people with mental health disorders within a defined geographical area. The study setting is one of the largest providers of mental healthcare in the UK, serving a population of approximately 1.4 million. It operates across a predominantly urban population with some semi‐rural areas located within its wider catchment. The NHS trust offers specialist inpatient and community mental health services, including community mental health teams, acute inpatient wards, rehabilitative settings, secure mental healthcare, intellectual disability care, and addiction services. Within the NHS trust, the new patient safety strategy and the PSIRF system were implemented in 2023. Previously, the SIF was in place.

### 2.3 Data Collection

Data was sourced from the patient safety department's safety reporting system who provided all reports completed across two 12‐month periods. The SIF/RCA reports were obtained from a 12‐month period prior to PSIRF implementation (January 2022 to January 2023). Level 1 and Level 2 SIF/RCA incident investigation reports were chosen for evaluation and comparison with systems‐based reports (PSLRs) following a discussion with the patient safety team. They were chosen as this level of report was thought to meet a similar incident threshold for investigation (based on complexity and severity) and be associated with an equivalent degree of quality/rigour in the incident investigation process to PSLRs.

The systems‐based reports (PSLRs) were sourced following PSIRF implementation from January 2024 to January 2025. A total of seven PSLRs were commissioned and completed within the 12‐month period across the mental healthcare divisions in the organisation. For SIF/RCA reports (January 2022 to 2023) a total of 28 incident investigation reports were commissioned and completed. Of the 28 reports, 18 were level one reports and 10 were level two reports. For comparative analysis, seven SIF/RCA reports were randomly chosen from the 28 extracted using simple random sampling. The units of analysis were decided upon on the basis of informational needs, where the number of reports chosen was thought to provide a sufficient number of recommendations/actions/improvements from the investigations to allow for the research question to be answered with sufficient confidence [24].

### 2.4 Data Analysis

First, to grade the quality of the reports, the analysis was guided by a validated tool that is used to inform constructive feedback on the quality of reports, the Learning Response Review and Improvement Tool [5]. Two authors independently applied the tool to the safety reports. Each report was scored on the tool's eight questions with ‘little evidence’ scoring 0, 'some evidence' scoring 1, and 'good evidence' scoring 2. Descriptive analysis was undertaken to compare total score and individual item scores between RCA and system‐based reports. An independent t‐test assuming unequal variances was used to analyse mean differences.

Second, we used inductive and deductive qualitative content analysis to analyse the investigation reports. Two authors initially familiarised themselves with the investigation reports. Each free‐text action, recommendation, and improvement within the report was initially extracted into a table. The manifest data was fully anonymised to exclude any patient, staff and team information. If the action, recommendation or improvement contained more than one solution this was taken as an additional code(s) for the next steps to be applied to.

Research has previously categorised safety actions, recommendations and improvements into ‘solution types’ [25, 26, 27]. We used this term within the evaluation for consistency with prior research. We developed codes deductively from solution types identified from prior research, applying these to the units extracted from the reports. For example, the final code ‘training’ was deductively coded from prior research that classified solution types of ‘staff training’, ‘training’, and ‘training and education’ [25, 26, 27]. Where data could not be deductively classified to pre‐existing codes, the data was inductively coded to exemplar solution types identified from the content analysis. Material SB shows the deductive and inductive codes developed through the analytic process and used in the analysis. Table 2 shows the final coding.

**Table 2:** Reported Solution Types between root‐cause analysis investigation reports under the Serious Incident Framework (SIF/RCA) and systems‐based incident investigation reports (PSLR) under the Patient Safety Incident Response Framework.

Solution category	Solution type	Frequency (*n/%*)		Example(s)
		SIF/RCA (*n* = *57)*	PSLR (*n* = 136)	
Supervision and Training	Supervision	2/4%	3/2%	“Reflective supervision to be completed with practitioners involved”
	Training	4/7%	13/10%	“Specific training on risk and victim safety to be requested from XX for XX Service Line”
Policies and Procedures	Update procedures/policy	3/5%	25/18%	“Update the XX Standard Operating Procedure to ensure there is a pathway included for difficult to engage patients”
	Develop procedures/policy	1/2%	2/1%	“Creation of a Divisional access SOP”
	Implement system process	6/11%	6/4%	“The Trust to develop a standardised process around the use of safety huddles”
	Monitoring adherence to national guidance	0/0%	3/2%	“Divisional management of NICE guidance to continue and to be enhanced by re launch of Divisional NICE and Audit Group”
Checking or improving staff performance	Individual competence	0/0%	5/4%	“Professionals should demonstrate in‐depth professional curiosity”
	Reinforce policy/quality standard	9/16%	26/19%	“Staff should ensure they record all clinical discussions within the patient record relating to patient care, including the rationale for the decisions made”
	Evaluate current practice/standard	2/4%	2/1%	“Trust to consider evaluation of current approaches to engagement of those newly diagnosed with ASC to explore their effectiveness and inform any necessary changes needed to maximise engagement, including the use of digital technologies”
	Task compliance check	2/4%	15/11%	“Service Line Observation Audit to be developed to monitor compliance with Supportive Observation Policy”
Working across organisational or team boundaries	Improving pathway processes and information sharing within and between services	5/9%	7/5%	“Implement a clinical governance oversight meeting between the providers to review processes, pathways and learning”
Resource availability and recruitment	Workforce change	2/4%	0/0%	“Workforce development review for XX to support embedding a dual diagnosis role and function”
Changes to administrative system	Forms/paperwork change	5/9%	4/3%	“To implement body mapping documentation as part of admission paperwork pack”
	IT structure change	3/5%	6/4%	“IT system to be updated to include MARAM section”
Physical environment change	Physical environment change	2/4%	0/0%	“Lockers procured and installed to enable safe storage of personal mobiles”
Commissioning Change	Commissioning change	2/4%	0/0%	“Budget to be transferred to XX to enable rapid ordering and delivery process”
Sharing of identified learning	Sharing across organisation	5/9%	11/8%	The Divisional Learning Newsletter will contain information for staff regarding increased risk during medication changes and will be shared across the Division”
	Sharing to individuals/teams	4/7%	8/6%	“Review findings to be shared with XX team to explore digital means”

The explicit, manifest data was extracted and coded to one or more solution type. The codes were conceptually defined by the content of the meaning units identified. From the extracted data and subsequent analysis, a set of solution type codes were generated and appraised. The two analysts reviewed and refined the solution type codes, with final oversight and agreement from the research team.

**Figure 1:**
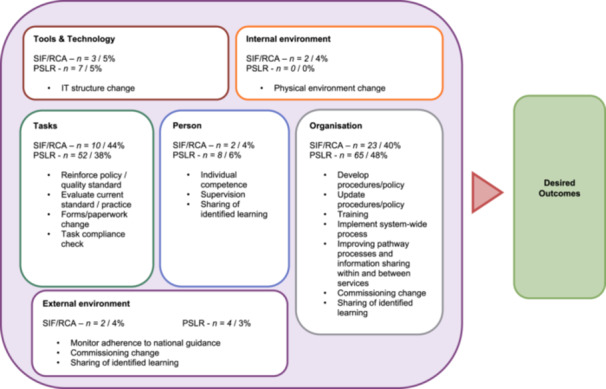
The frequency of system‐work factors found in extracted data, placed into the SEIPS model framework [21].

Next, each unit of extracted manifest data and its corresponding solution type was reviewed and organised into a category of work system factors as delineated through the SEIPS model e.g., organisation, tasks, external environment (Figure 1) [21, 28]. Descriptive statistics were used to investigate differences between the safety components identified, the solution categories, and the system‐work factors between RCA and systems‐based investigations.

Third, the solutions extracted from the reports were applied to the hierarchy of risk controls, a tool developed by the National Institute of Occupational Safety and Health to rank controls (i.e., safety action/recommendations/improvements) on effectiveness [22]. To improve applicability to healthcare research, studies have simplified the hierarchy into three tiers [29]:

- Elimination/substitution: Deemed the most effective, removing and/or replacing the hazard.
- Design controls: Also termed engineering controls, a mechanism to isolate people from the hazard. A focus may be on physical barriers, isolation, human factors, designing controls to improve safety elements independent of worker interactions.
- Administrative controls: The lowest tier of effectiveness, including training, updating policies/procedures, and other methods to shape people to take safer independent actions.

For our analysis, we placed solution types from the investigation reports into the three tiers of risk controls. Descriptive statistics were used to compare the solutions made between RCA and systems‐based investigations.

### 2.5 Quality Assurances

Two coders independently completed the first three steps on two RCA reports and two systems‐based reports to determine level of agreement. The Cohen's kappa statistical test was used to assess level of agreement. For the grading of the quality of reports using the tool the Cohen's kappa was 0.74 at a significance level of < 0.001, indicating a substantial/good level of agreement [30]. The two authors independently analysed the extracted manifest codes, analysing the solution types within the corresponding system‐based factor and effectiveness grading. The two authors met repeatedly to develop the coding framework and reach consensus agreements on the analytic process. As this level of agreement was reached and the analytic processes was solidified, one author proceeded to independently code the remaining reports, as detailed in the steps above. To ensure consistency over the remainder of the process, the authors met regularly to review progress and the codes identified. If there was uncertainty with the coding and/or an agreement was not reached, an additional member of the team was included in the discussions. The coding, allocation to system work factors and effectiveness rating was also reviewed and finalised with a director of patient safety and the team.

### 2.6 Ethics

The study used routinely accessible safety reports provided only to authors employed within the sponsoring organisation. The reports were fully anonymised and analysed by those authors within the service studied. The goal of the evaluation was to investigate the organisations shift to PSIRF, the report quality and the recommendations for learning for the organisation. As such, the study did not require ethical approval and was deemed to fall within the remit of a service evaluation by the sponsoring organisation (Registration: SE2025‐20).

## 3 Results

### 3.1 Investigations

Of the incidents that were chosen for further investigation under PSIRF, five (*n* = 5) were non‐accidental deaths, and two (*n* = 2) were homicides. Under the SIF, four reports were level 1 (comprehensive, internal/local) and three reports were level two (comprehensive, internal and external). The seven reports investigated incidents of non‐accidental deaths (*n* = 3), an unexpected death (*n* = 1), a deterioration of physical health (*n* = 1), a medication error (*n* = 1), and aggression (*n* = 1).

All the PSLR reports used the SEIPS model of systems‐based investigations. All the SIF reports used the RCA approach to incident investigation.

### 3.2 Investigation Report Quality

Table 1 shows the scoring breakdown of the application of the Learning Response Review and Improvement Tool to SIF/RCA reports and PSLRs [5]. This graded the quality of the investigation report against priorities and standards set out for the completion of systems‐based incident investigations.

### 3.3 Solution Type Coding and System Categorisation

A total of 192 recommendations/improvements/actions were extracted from the investigation reports. There were 135 recommendations from PSLRs and 57 SIF/RCA recommendations. A total of 19 solution type codes were identified from the recommendations, which were categorised into 9 solution categories. Table 2 displays the solution categories and type, with the frequency at which they were found in both SIF/RCA and PSLR reports.

Figure 1 displays the frequency of system‐work factors found in extracted data, placed into the SEIPS model framework [21]. This figure displays the breakdown of systems work factors more clearly, with examples of solution type codes detailed within each system work factor.

The most frequent solution category for both incident approaches was to check or improve staff performance. This included the most common solution type for both investigation approaches, to reinforce a policy or expected quality standard to staff completing a task or tasks. This was followed by an action to review/update an organisational policy/procedure for systems‐based investigations. Implementing a system‐wide process was the second most common solution type for SIF/RCA reports.

Organisational system factors were the most frequently observed from systems‐based investigations whilst task system work factors were most frequent for SIF/RCA reports. Examples of organisational system factors were administrative controls to update policies or procedures. Task‐based system work factors included a recommendation to reinforce the expected standards to the healthcare services. The evaluation found limited system work factors that led to physical environmental changes or changes to tools and technology within the mental healthcare setting.

An exemplar code and solution type unique to this evaluation was the sharing of identified learning, either widely across the system or to more local individuals/teams. The system factor category was aligned to the function or action associated with the sharing of learning, for example sharing learning across the organisation broadly or to evaluate or reinforce a task standard. The communication of a focussed review or action may have been linked to further learning or improvement action downstream of the immediate incident investigation action. For PSLRs fifteen (*n* = 15/79%) were focussed sharing linked to further safety review or action, and for SIF/RCA two (*n* = 2/22%) were focussed sharing.

### 3.4 Effectiveness of Recommendations

Each unit of extracted data and solution type were graded through the hierarchy of risk controls [22]. The most common for PSLRs was administrative controls (*n* = 121/89%), followed by design controls (*n* = 14/10%), and then elimination/substitution (*n* = 1/1%). The most frequent for SIF/RCA reports was administrative controls (*n* = 47/82%), followed by design controls (*n* = 8/14%), and then elimination/substitution (*n* = 2/4%). Examples of elimination/substitution within the evaluation included an updated policy and physical environment change to eliminate the use of personal mobile phones by staff. For design controls, an example including the implementation of a system‐wide process of a red‐amber‐green tool for escalation of risk.

## 4 Discussion

### 4.1 Main Findings

This evaluation examined whether a shift in safety strategy is genuinely visible in practice by assessing the learning, actions and improvements generated by incident investigations in one organisation. Uniquely, we did this through an at‐scale, within‐organisation comparison that combined structured quality grading with a systematic analysis of solution type, allowing a like‐for‐like assessment of what changes when practice moves from RCA to systems‐based investigation. Our findings show that the PSIRF shift is being realised. The reports showed clear improvements in safety‐related content, language, and underlying assumptions as the organisation moved from RCA style report to systems‐based investigation reports. Systems‐based reports had evidence of stronger compassionate involvement of those affected, clearer systems thinking, and more systems‐focused, evidence‐based and collaboratively developed safety actions. This is important because it demonstrates that the strategic shift in safety is being enacted in practice to a degree, with some observable evidence of changing conceptual thinking and investigative orientation.

In particular, the patient safety strategy emphasised engagement with staff, families, patients and carers, and our findings show an increase in compassionate involvement or even the possibility of involvement [31]. By design, PSIRF promotes involvement, and this evaluation provides quantitative evidence that such involvement is now more consistently embedded within investigations. Further ethnographic and qualitative enquiry will be important to understand how change is experienced by those involved and how it shapes service learning and improvement over time [32]. Both RCA and systems‐based investigations showed some evidence of counterfactual reasoning and the use of blame‐oriented language. Although mean scores improved under systems‐based investigations, these differences were not as large as other scoring domains. This may reflect the relative recency of PSIRF adoption and the developmental journey of investigators as they build confidence with systems thinking. It may also reflect wider organisational pressures and interdependencies that shape what investigations are expected to produce. This interpretation is consistent with qualitative evidence from mental healthcare showing that, even after a shift to system‐based investigations, elements of the wider safety system can change expectations and functions in the incident response pathway in ways that pull solutions back towards patterns more typical of RCA [15].

Our study showed that systems‐based approaches to investigation were consistently used under the new safety strategy, resulting in a higher quantity of solution types overall and more organisation system factors. Previous research evaluating recommendations from RCA reports noted that finding recommendations was more difficult than finding problems [27, 33]. It is possible that systems‐based investigations were better at finding solutions than the RCA approach. However, it may have been influenced by the nature of the incidents investigated under PSIRF and the SIF. The change in safety strategy granted organisations greater agency over incident selection for in‐depth investigations, where the previous SIF/RCA approach was criticised for creating an industry of investigations that lacked evidence of effectiveness [14]. This change may have improved resources for individual investigations, improving opportunities to develop solutions. It may have also resulted in different incident types, our study showing that all PSIRF investigations were undertaken on non‐accidental deaths and homicides, whilst SIF/RCA were wider reaching. The incident type may influence the quality of the investigation, as well as frequency and nature of the recommendations that are found.

The Health Services Safety Investigations Body recently published an exemplar investigation report based on an incident of attempted suicide while under the care of community mental health services. This report used the patient safety incident investigation template to support NHS organisations to increase their learning about systems‐based investigations and how PSIRF tools can be used to improve them [34]. The exemplar report has similarities to that observed in our analysis. There is clear, effortful involvement of service users, carers and family, a distinct shift observed in our analysis. Both the exemplar report and the PSIRF investigations show evidence of qualitative data gathering to form a balanced systems‐focussed narrative understanding of events.

Observed differences included the methods used to help organise, understand and analyse the gathered information in the exemplar report. The links between the narrative understanding, the development of themes to understand and explain why the incident occurred (findings, contributory factors), and the areas of improvement were clearly outlined in the exemplar report, with evidenced use of PSIRF tools to inform the investigation. In comparison, the PSIRF investigations analysed in our evaluation referenced these methods to help organise, understand and analyse information gathered, however, consistent, transparent, completion of a range of methods was unclear. Completion of a range of methods (e.g., inputting gathered information into a SEIPS framework and stakeholder map) may result in the development of more systems‐focussed safety actions.

### 4.2 Implications for Policy and Practice

For those conducting investigations and shaping recommendations, an improved understanding of *why* clinical decisions are made is an important first step [35]. Prior research suggests that incident investigations predominantly identify human error within the proceduralised clinical workflow, expecting the person to complete a task through adherence to organisational policies and assumed knowledge, or ‘*work‐as‐imagined’* [36]. An emphasis on ‘*work‐as‐imagined’* may mean incident investigations lean towards administrative, compliance‐dependent controls, as shown in our study and prior research [37, 38]. By focussing on ‘*work‐as‐imagined*’ the investigation may miss ‘*work‐as‐done*’. Investigating ‘*work‐as‐done’* includes identifying the dynamic circumstances and variability of real‐world clinical practice, such as the competing and contextual factors influencing why a clinical decision was made or the habitual activities of clinicians [36]. Conducting incident investigations with this in mind will prevent the recurrence of recommendations that attempt to reinforce a prescribed ideal (e.g., review and enhance policies and procedures, update training). It will hopefully identify why the ‘*work‐as‐done’* was not performed as imagined, allowing organisations to actively and constructively detect unwanted variability and identify areas for improvement [39].

Previous research has shown that following the identification of contributory factors and recommendations, there is limited evidence of feedback, and a lack of double‐loop learning needed for sustained improvements in safety [40]. In our study, weaker administrative recommendations remained common despite the shift to PSIRF, with training and the sharing of findings a common solution type. In contrast to SIF/RCA, the sharing of identified learning under PSIRF was more likely to be linked to further action and completion of a feedback loop. When further learning is linked, this needs to be shared in a way where it can be repositioned as a social deconstruction and reconstruction of knowledge [31, 41]. This may include a collaborative examination of the systems and processes to understand how care was delivered and how the care could be shaped, hopefully using investigation recommendations informed by prior investigatory work bridging the gap between *‘work‐as‐imagined’ and ‘work‐as‐done’* [36, 39]. Engaging the workforce in processes to address this gap has demonstrated positive influences on patient safety and job satisfaction [39]. It also holds greater promise that more complex, harder to identify, system‐level errors could be iteratively identified and addressed. This may in turn result in the development and implementation of stronger controls or barriers, rather than the weaker controls often tolerated in healthcare. Fostering meaningful collaborative involvement and learning may also support in reducing feelings of blame.

### 4.3 Implications for Research

Previous research has shown a disconnect between the contributory factors of an incident and the nature of solutions, suggesting a mismatch between incident investigation and future prevention [25]. The analysis of both contributory factors and recommendations has often been overlooked in previous research and is an area for future research to build an understanding of the pathway to develop systems‐based recommendations. Further studies on the use of the system‐based analytic tool of choice, the System Engineering Initiative for Patient Safety (SEIPS) framework, in mental healthcare is needed, where a recent review found only one study has applied the SEIPS model to a mental health setting [7, 42].

To grade effectiveness, we used the Hierarchy of Risk Controls [22]. However, alternative classification systems could have been used, such as the US Department of Veteran Affairs' recommendations effectiveness criteria [27, 36]. Regardless, these classification systems prioritise higher‐tier interventions for their reliability in modifying the system. Discovering these types of controls can be challenging in mental healthcare, with added complexities including the nature of incidents, the notion of risk, service user related factors, and the duality of interventions to manage risk [15, 43]. Identifying stronger controls to isolate or remove patients from these risks are not theoretically impossible. For example, installation of door top alarms to eliminate a ligature point. An issue arises from the transposition of design/engineering controls, which are more suited to stable hazards and standardised processes, into mental health's complex adaptive system, where hazards are latent and emergent, professionals exercise judgment, and care is co‐produced with agentic service users. Future research on how best to equip incident investigators with an understanding of the complex sociotechnical system is required. A focus on identifying ‘*work‐as‐done’* both in practice, as described above, and in empirical studies will support the development of this understanding.

### 4.4 Strengths and Limitations

A limitation of this review is that it took place within one organisation with a small sample size of reports. Although a large quantity of coded recommendations was identified, a larger sample size may have revealed a greater range of incident types, solution categories, and differences between investigation approaches. Our evaluation showed that SIF/RCA investigations had a greater range of incident types, where systems‐based approaches had only investigated serious incidents of non‐accidental deaths or homicide. Completing the evaluation as a robust research study across more than one NHS trust may have discovered differences between varied clinical services and safety teams. This evaluation sourced reports in the early stages following PSIRF implementation, meaning that the quality of systems‐based investigations may be impacted by limitations in implementation and experience. Another limitation is that our study used the Learning Response and Review tool, a tool designed to evaluate systems‐based approaches to investigation [5]. The tool was designed to evaluate systems‐based approaches, with questions within the tool focussed on the quality of systems‐based investigations, making it less applicable to grading RCA reports. However, we opted to use the tool to assess if the organisation had met the quality standards and expectations of the new safety strategy. Additionally, the tool itself has had its content validated by its users [5]. However, formal dissemination with evidence of validity and interrater reliability cannot be sourced, making it less reliable for use in research.

## 5 Conclusion

The evaluation provides important insights into the nature of solutions from RCA and systems‐based investigations. The evaluation showed that some of the key tenets of the change to systems‐based investigations are being realised, including patient/carer/family involvement and systems‐focussed solutions. However, solution types classified as weak administrative controls were commonly found across both systems‐based and RCA investigations. This evaluation is an important initial exploration of the change in incident investigation approaches, which will help guide policy, practice and research towards more effective, evidence‐based, systems focussed solutions in patient safety.

## Author Contributions

Author Alexander Challinor conceived and designed the study. Data was collected and analysed by authors Alexander Challinor and Sahil Bhandari. All authors reviewed and finalised the outcomes from the analysis. Author Alexander Challinor drafted the manuscript. All authors made critical revisions and contributed important intellectual content to the final version. Author Alexander Challinor is the guarantor.

## Conflicts of Interest

Oladayo Bifarin is a National Institute for Health and Care Research Leader. The views expressed in this article are those of the author(s) and not necessarily those of NIHR or the Department of Health and Social Care. All other authors have no competing interest to declare.

## Supporting information

Supporting File 1

Supporting File 2

## Data Availability

Permission to access our study data is only granted to researchers in the study team. The data are not publicly available due to ethical restrictions e.g., their containing information that could compromise the privacy of patients.
