# Supplementary material for: From Root Cause Analysis to Systems Thinking: A Comparative Content Analysis of Patient Safety Incident Investigation Reports in Mental Healthcare
Source: J Eval Clin Pract. 2026 Jun 7;32(4):e70495. doi: 10.1111/jep.70495 (PMC13242895; doi:10.1111/jep.70495)
Supplement: Supplementary file 2 — Supporting File 2 [file JEP-32-0-s001.docx]

**Supplemental Material**

The table below shows solution types from prior research. Following our analysis, final codes were chosen, either maintained or adapted from deductive analysis. For data that did not fit with previous solution types, an exemplar code was created from inductive analysis.

| Solution Type/Category Codes | | | |
| --- | --- | --- | --- |
| Averill et al., 2025. (1) | Kellogg et al., 2017. (2) | Hibbert et al., 2018.(3) | Final Coding |
| Streamlined system for managing paper documentation | Forms and paperwork change | Enhanced documentation, communication | Forms / paperwork change |
| - | Compliance check | Audit undertaken  Checklist / cognitive aids | Task compliance check |
| Procedural changes | Policy change | Policy/ guideline / documentation, etc. review/enhancement | Update procedures/policy |
| Procedural changes | Policy change | New procedure / memorandum / policy | Develop procedures/policy |
| Reinforcement of existing policy | Policy reinforcement | - | Reinforce policy/quality standard |
| Staff recruitment or new care pathway to address gap in service offer | Institutional change | Review rostering / appropriateness of staff mix  Increase in staffing / decrease in workload | Workforce change  Commissioning change |
| Staff training | Training | Training and education | Training |
| Implementation of systems for management | Process change | Simplify Process | Implement system process |
| IT structure change | IT structure change | Software enhancements / modifications | IT structure change |
| Supervision | Counselling | - | Supervision |
| - | Physical environment change | Architectural/physical plant changes | Physical environment change |
| Improving information sharing within and between services | - | Standardised communication tools | Improving pathway processes and information sharing within and between services |

**Exemplar codes from inductive coding, unique to this study.**

- Evaluate current practice / standard
- Individual competence
- Sharing of identified learning ---> Sharing across organisation, Sharing across teams
- Monitoring adherence to national guidance

**References for supplemental material table:**

1. Averill P, Sevdalis N, Henderson C. Patient safety incidents within adult community-based mental health services in England: A mixed-methods examination of reported incidents, contributory factors, and proposed solutions. Psychological Medicine. 2025;55:e8.

2. Kellogg KM, Hettinger Z, Shah M, Wears RL, Sellers CR, Squires M, et al. Our current approach to root cause analysis: is it contributing to our failure to improve patient safety? BMJ quality & safety. 2017;26(5):381-7.

3. Hibbert PD, Thomas MJW, Deakin A, Runciman WB, Braithwaite J, Lomax S, et al. Are root cause analyses recommendations effective and sustainable? An observational study. International Journal for Quality in Health Care. 2018;30(2):124-31.
